# Modern Operations for Cancer

**Published:** 1898-01-29

**Authors:** 


### Modern Operations for Cancer.

So much stress has been laid of late on the necessity for thoroughness in all operations for cancer, that much interest attaches to the very careful paper on immunity and latency after operations for reputed carcinoma of the breast, which was read by Mr. Marmaduke Sheild before the Royal Medical and Chirurgical Society on Tuesday. What he aimed at showing was (1) that the results of so-called incomplete operations are not so universally bad as is generally supposed; (2) that the progress of cancer is subject to curious vagaries, the disease remaining latent for extraordinary periods in many cases; and (3) that some cases in which life has been prolonged for very long periods have been associated with operations upon local recurrences. The history of the operative measures undertaken for the removal of cancer of the breast is of some interest, showing as it does how professional opinion on the subject has varied from time to time. It is a matter of general belief that the results of removal of the breast for carcinoma in the first half of this century, and indeed up to quite modern times were essentially bad, and, in fact, the views held by many of the early surgeons in regard to the utility of operation were pessimistic to a degree.

No doubt the unfortunate experience which led to such views was to a large extent due to the very incomplete form of operation which was then in vogue. A kind of revulsion had taken place against the old and very complete but barbarous method of sweeping off the entire mamma, skin and all, and freely cauterising the surface left. Surgeons had come round to an endeavour to obtain flaps, and to cover in the wound by skin, ignoring the fact that they were in too many instances actually shutting up cancerous foci in the wound, the growth in its totality never having been moved at all. Then came the modern method of complete removal, in regard to which many claims have been put forward that a complete cure has resulted. Now, in regard to these no one can study the results obtained by such men as Dennis, and Meyer, and Halsted, and those recorded by Watson Cheyne in his Lettsonian lectures, without seeing that the "complete" operation lessens the liability to local recurrence to a marked degree. The question remains, however, whether this result can properly be called a cure, and how far the non-recurrence is due to the completeness of the operation? Mr. Shield gives forty-four cases where no recurrence took place, although the patients lived after the operation for periods varying from six to twenty years. In some of these cases the operation was markedly incomplete. In one mammary substance was actually left round the tumour, and the enlarged glands which already existed in the axilla were not removed, yet when the patient died seven years later from cirrhosis no trace of cancerous disease was discovered.

In another group, 49 cases are given in which recurrence did occur at dates varying from the fourth to the twenty-fifth year after the operation. This group seems to us the most important of the two—on the one hand, because the ultimate recurrence is the strongest proof that we can well have of the malignancy of the growth; on the other, because the time which elapsed before return took place shows pretty plainly that non-recurrence for three years is not to be taken as proving a cure; and still more, because in some of these cases, notwithstanding their proved malignancy, and the removal of both the primary and secondary growths in what would now be called an incomplete manner, the patients ultimately remained free for many years.

The point which arises from a consideration of all these cases is that true as it is that very complete removal does very materially prevent local recurrence, there is some other influence at work, of which at present we know nothing, on which, as well as on the operation, depends the ultimate fate of the patient. Some cases, which have undergone the completest removal which modern surgery can accomplish, quickly recur; and others, in which no local recurrence takes place, die of metastatic deposits. Yet all the time a fair number in which the removal has been far from complete live on without recurrence, or even when recurrence has taken place time after time, and each time has been removed by operation, ultimately attain good health. What is this other influence—this general constitutional state which decides whether a cancer shall grow or shrivel? That is what we want to know.

